# Characterization of Colistin-Resistant *Escherichia coli* Isolated from Diseased Pigs in France

**DOI:** 10.3389/fmicb.2017.02278

**Published:** 2017-11-21

**Authors:** Sabine Delannoy, Laetitia Le Devendec, Eric Jouy, Patrick Fach, Djamel Drider, Isabelle Kempf

**Affiliations:** ^1^Agence Nationale de Sécurité Sanitaire de l'Alimentation, de l'Environnement et du Travail, Food Safety Laboratory, Université Paris-Est, Maisons-Alfort, France; ^2^Agence Nationale de Sécurité Sanitaire de l'Alimentation, de l'Environnement et du Travail, Ploufragan Laboratory, Ploufragan, France; ^3^Université Bretagne Loire, Rennes, France; ^4^Institut Charles Viollette, Université de Lille, Lille, France

**Keywords:** colistin resistance, virulence, *Escherichia coli*, pig

## Abstract

We studied a collection of 79 colistin-resistant *Escherichia coli* isolates isolated from diseased pigs in France between 2009 and 2013. We determined a number of phenotypic and genetic characters using broth microdilution to characterize their antimicrobial susceptibility. We performed pulse field gel electrophoresis (PFGE) to assess their genetic diversity and assign them to phylogroups. High-throughput real-time PCR micro-array was used to screen for a selection of genetic markers of virulence, and PCR and sequencing of the main recognized resistance genes allowed us to investigate the mechanisms of colistin resistance. Results showed that isolates belonged to several phylogroups and most had a unique PFGE profile. More than 50% of the isolates were also resistant to sulfonamides, trimethoprim, tetracycline, ampicillin or chloramphenicol. The *mcr-1* gene was detected in 70 out of 79 isolates and was transferred by conjugation in 33 of them, sometimes together with resistance to sulfonamides, trimethoprim, tetracycline, ampicillin, chloramphenicol, cefotaxime, or gentamicin. Mutations in the amino-acid sequences of proteins MgrB, PhoP, PhoQ, PmrB, but not PmrA, were detected in isolates with or without the *mcr-1* gene. More than one-third of the isolates harbored the F18, F4, *astA, hlyA, estI, estII, elt, stx*_2*e*_*, iha, orfA, orfB, paa, terE, ecs1763*, or *ureD* virulence markers. In conclusion, although most isolates had a unique PFGE profile, a few particular combinations of phylogenetic groups, virulence genes and mutations in the sequenced genes involved in colistin resistance were identified on a number of occasions, suggesting the persistence of certain isolates over several years.

## Introduction

*Escherichia coli* is one of the main pathogenic bacterial agents in pigs. Depending on its virulence factors, it can cause various diseases including mainly enteric diseases such as neonatal diarrhea, post-weaning diarrhea (PWD), and edema disease (ED), but also systemic infections such as septicemia, polyserositis, mastitis, and urinary tract infections (Fairbrother and Gyles, 2012). These diseases result in morbidity, mortality, and delayed growth (Fairbrother et al., 2005). In addition to hygiene and biosecurity measures and vaccination of sows or animals at risk, antimicrobials are often used to control the clinical outcomes of *E. coli* infections (Fairbrother et al., 2005). They are most commonly administered to pigs by the oral route, either in feed or in water, and digestive disorders are the first indications for antimicrobial use in pig production (Chauvin et al., 2002). The most frequently administered antimicrobials include polypeptides and aminoglycosides. Colistin (CST) is a polypeptide antimicrobial frequently used in pig production. In France in 2013, according to Hemonic et al. (2014, 2016), 80% of a representative sample of pig farms used polymyxins in post-weaning piglets, 42% in suckling piglets and 15% in fattening pigs. In 2010, the reported use was mainly related to the treatment of digestive disorders (Hemonic et al., 2014), and a study comparing usage in 2010 and 2013 revealed a significant reduction in use between those years (Hemonic et al., 2016).

Until late 2015, acquired resistance to CST in *Enterobacteriaceae* was thought to be exerted through chromosomal mechanisms including lipopolysaccharide (LPS) modifications such as the addition of 2-aminoethanol, 4-amino-4-deoxy-L-arabinose (L-Ara4N) or phosphoethanolamine (PetN), or other strategies such as efflux pump and capsule formation (Olaitan et al., 2014b). Modifications of lipid A in the LPS can be linked to mutations resulting in the constitutive activation of the two-component systems involving PhoP/PhoQ and PmrA/PmrB or to mutations or inactivation of the *mgrB* gene and subsequent negative feedback of the PhoP/PhoQ system. Other genes, such as *etk* coding for a tyrosine-kinase and *mgrR*, can also play a role in resistance to CST in *E. coli* through modifications of the negative charges of the LPS. In addition to chromosomal resistance, plasmid-mediated resistance can occur as described in China in 2015 (Liu et al., 2016). The first description of the plasmid *mcr-1* gene coding for a PetN transferase enzyme was rapidly followed by a myriad of reports showing the presence of the *mcr-1* gene on various plasmids in strains of human, animal or environmental origin in most parts of the world (Kempf et al., 2016). In 2016, the *mcr-2* gene, also coding for the addition of PetN to lipid A, was discovered in porcine and bovine *E. coli* strains in Belgium (Xavier et al., 2016b). Recently, a third mobile colistin resistance gene, *mcr-3*, was characterized in a porcine *E. coli* strain (Yin et al., 2017).

Several monitoring systems have been implemented in France to evaluate the susceptibility of bacteria isolated from animals to antimicrobials. They include the antimicrobial susceptibility testing of bacteria isolated from healthy animals at the slaughterhouse as part of European surveillance schemes (EFSA-ECDC, 2015), the *Salmonella* network dedicated to the monitoring of *Salmonella* isolates from the French agricultural food sector, and the surveillance of the susceptibility of isolates from pathological cases, through the RESAPATH network (https://www.resapath.anses.fr/). In 2009–2011, this latter network revealed 55–60% of *E. coli* isolates with CST inhibition diameters of 18 mm or more [i.e., CST-susceptible (CSTS) isolates] in pigs; however, more recent studies on isolates from 2011 to 2015 have shown susceptibility prevalence levels higher than 70% (Gay et al., 2016). CST resistance remains an issue to be addressed because CST is becoming a last-resort antimicrobial for human health. The aim of this study was therefore to characterize the CST-resistant (CSTR) isolates available in the RESAPATH network to evaluate their diversity and resistance mechanisms. Thus, we collected the CSTR isolates previously isolated and tested by local veterinary laboratories.

## Materials and methods

### Isolates

On a voluntary basis, diagnostic laboratories sent isolates isolated from diseased pigs, when the inhibition zone diameter according to the NFU-47-107 disk diffusion method (AFNOR, 2012) was <17 mm. Upon arrival, the isolates were coded according to the year of reception in our laboratory (two first numbers of the code), and their order of arrival. The isolates collection included 81 isolates, mainly from farms in northwestern France. Eighteen isolates were isolated in 2009, 27 in 2010, 17 in 2011, 11 in 2012 and 8 in 2013. Data related to the status of the animals were exceptionally available for 76 isolates: 11 were obtained from suckling piglets, 63 from post-weaning piglets, 1 from a fattening pig and 1 from a sow. The type of sample was known for 68 isolates: 63 were of intestinal or fecal origin, 2 from animals with septicemia, 1 from the nervous system, 1 from a lymphatic node, and 1 from urine. In addition, nine isolates obtained from diseased pigs between 2004 and 2015, and for which inhibition zone diameter was higher than 17 mm were included for the study of the sequences of the *pmrA, pmrB, mgrB, phoP*, and *phoQ* genes. Upon arrival at the ANSES laboratory, the identification of *E. coli* was checked by a PCR test based on the *uidA* gene (Bej et al., 1991).

### Genetic diversity of the isolates studied by PFGE and phylogroup determination

The diversity of the isolates was studied using pulse-field gel electrophoresis (PFGE) (Ribot et al., 2006) after digestion with the restriction enzyme *Xba*I. A similarity matrix was obtained on the basis of the Dice coefficients. The relationships between PFGE patterns were calculated by the unweighted pair group method with arithmetic mean (UPGMA) with confidence interval of 5% and the dendrogram representing the genetic relationships between the *E. coli* isolates was drawn using the Biogene package (Vilber-Lourmat, Marne-la-Vallée, France) as previously described (Marois et al., 2001). The phylogenetic groups of the isolates were determined according to Clermont et al. (2013).

### Antimicrobial susceptibility

The susceptibility of the isolates was then determined using the broth microdilution method on EUVSEC plates (Sensititre, ThermoFisher Scientific, Dardilly, France). Briefly, 50 μL of a suspension containing approximately 10^5^ colony forming units (CFU)/mL were deposited in each well of the plate. The plates were incubated for 18 to 24 h at 35°C before reading. The *E. coli* strain ATCC25922 was used as a control for each series. The isolates were then classified as wild-type (susceptible) or non-wild-type (resistant) according to the epidemiological cut-offs proposed by EUCAST (http://mic.eucast.org/Eucast2/). The tested antimicrobials were colistin (CST), sulfamethoxazole (SMX), trimethoprim (TMP), tetracycline (TET), tigecycline (TGC), nalidixic acid (NAL), ciprofloxacin (CIP), ampicillin (AMP), cefotaxime (CTX), ceftazidime (TAZ), meropenem (MER), chloramphenicol (CHL), gentamicin (GEN), and azithromycin (AZI). For isolates that appeared resistant to cephalosporins, the presence of extended-spectrum beta-lactamases was investigated by agar diffusion, using disks of CTX and amoxicillin-clavulanic acid. In cases of synergy between CTX and amoxicillin-clavulanic acid disks, the presence of *bla*_CTX−M_ genes was screened using a PCR protocol (Woodford et al., 2006).

### Mechanisms of resistance to colistin

Transfer of resistance to CST was tested by conjugation with CSTR isolates as donors and rifampicin-resistant *E. coli* J5 or rifampicin- and GEN-resistant *E. coli* UA6190 as recipient strains, using agar media supplemented, respectively, with rifampicin (150 mg/L) and CST (8 mg/L), or supplemented with rifampicin (150 mg/L), GEN (4 mg/L), and CST (8 mg/L). Appropriate controls were included to check the rifampicin–susceptibility of the donor isolates. The transconjugant profiles obtained using PCR targeting enterobacterial repetitive intergenic consensus sequences (ERIC-PCR) (Rivera et al., 1995) were compared with those of the recipient and the donor strains and the presence of the *mcr* genes in the transconjugants was checked by PCR as described below. The minimum inhibitory concentrations (MICs) of the transconjugants were studied using the broth microdilution method as previously described. The replicon-types of the isolates and their transconjugants were determined (Carattoli et al., 2005; Poirel et al., 2016).

To investigate the mechanisms of resistance to CST, DNA was prepared from bacterial cultures with an InstaGene matrix (BioRad). The *mcr-1* gene was screened using PCR with the primers defined in Liu et al. (2016) and the EU-RL protocol for antimicrobial resistance (https://www.eurl-ar.eu/CustomerData/Files/Folders/21-protocols/278_mcr-multiplex-pcr-protocol-v2-oct16.pdf). Amplified products were detected after agarose gel electrophoresis. The *mcr-1* control strain was kindly provided by A. Perrin-Guyomard, ANSES Fougères (Perrin-Guyomard et al., 2016). The *mcr-2* gene was screened using PCR (Xavier et al., 2016b) and the control strain containing pKP37-BE was kindly provided by S. Malhotra-Kumar (University of Antwerp, Netherlands). The *mcr-3* PCR was as described by Yin et al. (2017) and the *mcr-3*-positive Singapore strain was kindly offered by R. S. Hendriksen (National Food Institute, Lyngby, Denmark).

The *pmrA, pmrB, mgrB, phoP, phoQ*, and (when present) *mcr-1* genes were sequenced for the 81 CSTR or *mcr-1* positive isolates and for the 9 CSTS *E. coli* isolates obtained from diseased pigs from the RESAPATH network. Sequencing of the *pmrA, pmrB, mgrB, phoP, phoQ*, and *mcr-1* genes was performed using an Illumina MiSeq platform (Illumina) according to the manufacturer's instructions. The raw reads were assembled with the CLC Genomics Workbench version 7.5.1. The primers used for targeted sequencing are described in Table S1.

Sequences were used to query GenBank using the Basic Local Alignment Search Tool (BLAST; http://blast.ncbi.nlm.nih.gov/Blast.cgi). The coding sequences were then searched using http://insilico.ehu.es/ and compared using CLUSTALW multiple alignment (https://npsa-prabi.ibcp.fr/cgi-bin/npsa_automat.pl?page=/NPSA/npsa_server.html) (Combet et al., 2000) and http://www.phylogeny.fr/ (Dereeper et al., 2008, 2010).

### Virulence factors

Isolates were cultivated on Mueller-Hinton medium containing sheep blood for 18–24 h at 37°C to detect hemolysis. DNA from pure cultures was extracted as described previously and tested by PCR for various genetic markers. A high-throughput real-time PCR micro-array (Tseng et al., 2014) was used to screen for a selection of genes, mainly related to gastrointestinal pathogenic *E. coli* such as Shiga-toxin producing *E. coli* (STEC), enterohemorrhagic *E. coli* (EHEC), and enteropathogenic (EPEC) *E. coli* (Fairbrother et al., 2005; Fairbrother and Gyles, 2012; Tseng et al., 2014; Baranzoni et al., 2016). The *wecA* gene was included as a positive *E. coli* control. The genetic markers included genes coding for O antigens (O8abc, O139, O141, and O149), F antigens (F4, F5, F18, and F41), toxins [*estI, estII, elt, stx*_2*e*_, *hlyA* (alpha hemolysin), *astA* (EAST1, heat-stable cytotoxin associated with enteroaggregative *E. coli*), *ehxA* (enterohemolysin)], adhesins [*eae* (intimine), *lpfA*_O113_ (long polar fimbriae), *lpfA*_O157_, *iha* (iron regulated gene A homolog), *orfA, orfB* (adhesin involved in diffuse adherence AIDA), *paa* (porcine attaching and effacing associated adhesion), *saa* (STEC agglutinating adhesion), *efa1* (EHEC factor for adherence), *bfpA* (bundle forming pilus), *aggR* (major fimbrial subunit of aggregative adherence fimbriae)], iron acquisition systems [*irp2* (iron repressible protein), *fyuA* (ferric *Yersinia* uptake)] and other genes such as *ureD* (urease transporter), *terE* (tellurite resistance), *epeA* (serine protease), *ecf1* (enzyme that enhance bacterial membrane structure), *nleB* (non-LEE-encoded type 3 secretion system), *ecs1763* (hypothetical protein, putative gene marker for EHEC), and *ipaH* (invasion plasmid antigen).

## Results and discussion

### Phylogenetic groups and PFGE profiles

PCR confirmed that all isolates belonged to the *E. coli* species. The new Clermont *E. coli* phylotyping method showed that isolates belonged to groups A (51 isolates, 63%), D (14 isolates, 17.3%), B1 (9 isolates, 11.1%), E (4 isolates, 5%), B2, C and F (one isolate each, 1.2% each).

Four isolates (UB 09-108, UB 10-019, UB 10-380, and UB 10-384) could not be analyzed using PFGE, whereas a PFGE profile was obtained for the other 77 *E. coli* after digestion with *XbaI*.

Upon visual inspection, most isolates displayed a unique profile (Figure S1), and isolates isolated from 2009 to 2013 were evenly distributed in the dendrogram. Interestingly, isolates shared at least 66% homology, but only four pairs of isolates shared a common PFGE profile (09-242 and 10-004; 10-157 and 10-184; 13-048 and 13-064; and 11-472 and 13-075). Both isolates of the first and the second pairs belonged to phylogroups D and A, respectively, but the other two pairs differed in phylogroups (11-472 belonged to group A, 13-075 and 13-064 to group E, and 13-048 to group D).

### Resistance to antimicrobials

Disk diffusion is not recommended for colistin, and laboratories of the RESAPATH network are now encouraged to determine the colistin MIC and perform a Colispot (Jouy et al., 2017). However, during several years, disk diffusion was used as a screening test. The 9 isolates with inhibition zone diameter higher than 17 mm were inhibited by 2 or <2 mg/L on Sensititre plates. The determination of the MIC of CST showed that 79/81 isolates with inhibition zone diameter <17 mm were indeed resistant because their MIC were 4 mg/L (27 isolates), 8 mg/L (34 isolates) or 16 mg/L (18 isolates). Two isolates (10-322 and 10-325) with inhibition zone diameter <17 mm were inhibited by 2 mg/L on Sensititre plates, confirming the poor reliability of the disk diffusion method. The *mcr-1* gene was detected in the 2 isolates with CST MIC of 2 mg/L (isolates 10-322 and 10-325, thus *mcr-1*-positive CSTS isolates) and in 70 out of 79 isolates with MIC of 4 to 16 mg/L; it was absent in the 9 CSTR isolates with inhibition zone diameter <17 mm and MICs from 4 to 16 mg/L and in the 9 isolates with inhibition zone diameter higher than 17 mm (Table S2). The gene was detected in isolates isolated from 2009 to 2013, and in 43 out of 51 isolates of phylogenetic group A, in all isolates of groups D, B1, E, C, and F, but not in the unique B2 isolate. The *mcr*-*2* and *mcr-3* genes were never detected.

The percentages of resistance to other antimicrobials of the 79 CSTR isolates are presented in Table 1. All but one isolate were multi-drug-resistant (resistant to 3 different antimicrobial families or more). The most frequent resistance profiles were CST-SMX-TMP-TET-AMP-CHL-GEN (8 isolates), CST-SMX-TMP-TET–AMP-CHL (7 isolates), CST-SMX-TMP–TET-NAL-CIP-AMP-CHL-GEN (6 isolates), CST-SMX-TMP-TET-CHL, or CST-SMX–TMP-TET (5 isolates each). One isolate (10-413) was resistant to all tested antimicrobials except MER, TGC and GEN. Among the 15 isolates that were resistant to CTX, 8 showed synergy between CTX and amoxicillin-clavulanic acid according to agar diffusion, and the *bla*_CTX−M−1_ gene was detected in all of them. Among the four pairs of isolates producing the same PFGE profile, only isolates 13-048 and 13-064 shared the same antimicrobial resistance profile (COL-SMX-TMP-TET-AMP-CTX-TAZ) and both had the *bla*_CTX−M−1_ gene.

**Table 1:** Non-wild-type and resistant *E. coli* isolates (among the 79 CSTR isolates).

**Antibiotic**	**CST**	**SMX**	**TMP**	**TET**	**TGC**	**NAL**	**CIP**	**AMP**	**CTX**	**TAZ**	**MER**	**CHL**	**GEN**	**AZI**
ECOFF (mg/L)	2	64	2	8	0.5	16	0.06	8	0.25	0.5	0.125	16	2	16^*^
Number of non-wild-type^**^ (%)	79 (100%)	78 (99%)	73 (92%)	75 (95%)	1 (1%)	26 (32%)	26 (32%)	55 (70%)	15 (18.5%)	13 (16%)	0 (0%)	51 (65%)	39 (49%)	8 (10%)
EUCAST Breakpoint	2	ND	4	8	2	ND	1	8	2	4	8	8	4	ND
Number of resistant^***^ (%)	79 (100%)	ND	73 (92%)	75 (95%)	0 (0%)	ND	6 (7%)	55 (70%)	8 (10%)	2 (2%)	0 (0%)	52 (66%)	38 (48%)	ND

* The tentative epidemiological cut-off (ECOFF) for EFSA was used (16 mg/L);

** Non-wild-type: MIC > ECOFF;

*** *Resistant: MIC> Breakpoint. Sulfamethoxazole (SMX), trimethoprim (TMP), ciprofloxacin (CIP), tetracycline (TET), meropenem (MER), azithromycin (AZI), nalidixic acid (NAL), cefotaxime (CTX), chloramphenicol (CHL), tigecycline (TGC), ceftazidime (TAZ), colistin (CST), ampicillin (AMP) and gentamicin (GEN)*.

### Conjugative transfer of the *mcr-1* gene

Although two recipient strains were used, the conjugative transfer of the *mcr-1* gene was successful for only 33 of the 70 *mcr-1*-positive CSTR and one of the two *mcr-1*-positive CSTS isolates. Absence or low frequency of transfer of the *mcr-1* gene has already been reported (Anjum et al., 2016); however, for other authors, the *mcr-1* containing plasmids are highly mobile (Quesada et al., 2016). The 34 transconjugants were also frequently resistant to SMX (20 transconjugants), TMP (20 transconjugants), TET (19 transconjugants), CTX (2 transconjugants), CHL (11 transconjugants), AMP (12 transconjugants), and GEN (8 transconjugants). Such co-transfer of resistance has been reported previously (Poirel et al., 2017) and the presence of various resistance genes in the same plasmid may promote the selection of CST resistance by other frequently used antimicrobial agents as tetracyclines or sulfonamides. The co-transfer of CTX resistance was observed in only one isolate, contrary to the frequent association of CST resistance and extended-spectrum cephalosporin-resistance in strains from calves (Haenni et al., 2016).

The following plasmid replicon types were detected in the transconjugants: IncI1 (19 transconjugants), IncP (14 transconjugants), IncX4 (10 transconjugants), IncI2 (5 transconjugants), IncN (3 transconjugants), IncF (3 transconjugants), and IncFIC (1 transconjugant). Some transconjugants harbored several replicon types such as the combinations (I1, I2), (I1, FIC), (I1, P), (I1, X4), (I2, P), (N, X4), (P, X4), (I1, N, I2), (I1, P, F), and (I1, N, P, F). The CTX-resistant transconjugant harbored IncI1 and IncX4 replicons. The *mcr-1* gene has already been reported in plasmids belonging to different replicon types (IncI2, IncHI1, IncHI2, IncFIB, IncFII, IncP, IncX4, and IncY) (Xavier et al., 2016a; Zhi et al., 2016; Zurfluh et al., 2016; Zhang et al., 2017) but has also been detected, albeit more rarely, on the *E. coli* chromosome (Veldman et al., 2016). In this study, transfers occurred in only 34 of the 72 *mcr-1*-positive isolates, but no attempt was made to further determine the genetic location of this determinant.

### Sequencing results

The sequencing and virulence results are presented in Tables 2–4. Sequencing confirmed the presence of the *mcr-1* gene in 72 *E. coli* isolates (Tables 2, 4). For all 72 isolates, the nucleotide sequence was 100% identical to the sequence of the entire *mcr-1* gene of *E. coli* strain SHP45 plasmid pHNSHP45.

**Table 2:** Mutations of the MCR-1, MgrB, PhoP, PhoQ, PmrA, and PmrB amino-acid sequences observed only in the 79 colistin-resistant (CSTR) or the 2 *mcr-1*-positive colistin-susceptible (CSTS) isolates.

**Target (number of full sequences obtained)**	**Amino acid mutations (number of CSTR or *mcr-1*-positive CSTS isolates)**	**GPR in which the gene or mutation is present (see Table 4)**	**Previously described similar mutations**
MCR-1 (72 isolates)	–	GPR 1, 2, 4, 6–11, 14, 15, 17, 18	
MgrB (79 CSTR, 2 *mcr-1*-positive CSTS and 9 *mcr-1*-negative CSTS isolates^&^)	V8A (1 CSTR) Q33R (3 CSTR)	GPR 3 GPR 4	L8Y in CSTR *K. pneumonia*e^*^ (Olaitan et al., 2014a) D31N in CSTR *K. pneumoniae* ^*^(Olaitan et al., 2014a)
PhoP (79 CSTR, 2 *mcr-1*-positive CSTS and 9 *mcr-1*-negative CSTS isolates^&^)	V108M (28 CSTR) A182P (1 CSTR)	GPR 2 GPR 5	V108M frequent in GenBank, impact on CSTR unknown
PhoQ (79 CSTR, 2 *mcr-1*-positive CSTS and 9 *mcr-1*-negative CSTS isolates^&^)	L87P (1 CSTR) S138T (8 CSTR) A166V (1 CSTR) I175F (3 CSTR) E464D (29 CSTR and 1 *mcr-1*-positive CSTS isolates) A482T (29 CSTR and 1 *mcr-1*-positive CSTS isolates)	GPR 6 GPR 4, 8 GPR 9 GPR 10 GPR 2, 11 GPR 2, 11	Many detected mutations in *E. coli*, impact on CSTR unknown (Anjum et al., 2016) L96P, S174N, L348Q and G385S in CSTR *K. pneumoniae* ^*^(Olaitan et al., 2014b)
PmrA (78 CSTR, 2 *mcr-1*-positive CSTS and 9 *mcr-1*-negative CSTS isolates^&^)	–		
PmrB (79 CSTR, 2 *mcr-1*-positive CSTS and 9 *mcr-1*-negative CSTS isolates^&^)	E123D (1 CSTR) S138N (9 CSTR) T156A (1 CSTR) G160E (1 CSTR) V161G (5 CSTR) V351I (2 CSTR) A361V (1 *mcr-1*-positive CSTS isolate) Truncated protein: 5 amino acids (1 CSTR) or 149 amino acids (1 CSTR)	GPR 3 GPR 4, 7, 8 GPR 2 GPR 12 GPR 13, 14 GPR 3, 7 GPR 15 GPR 16, 17	E121A, S124P in CSTR *Salmonella*, impact on CSTR unknown (Anjum et al., 2016) T140P, T157P in K. pneumoniae^*^ (Olaitan et al., 2014b) V161L/M/G in *Salmonella* (Anjum et al., 2016) and V161G in *E. coli* (Quesada et al., 2015), impact on CSTR unknown

*GPR, Genetic profiles of resistance, see Table 4 which gives for each GPR the different modifications observed in the sequenced genes (the impact of the mutations detected in this study has not been demonstrated)*.

* *Shown to contribute to CST-resistance*.

& *The genes encoding MgrB, PhoP, PhoQ, PmrA and PmrB were sequenced for all isolates, and the results obtained for the 79 CSTR isolates and the two mcr-1-positive CSTS isolates were compared to those obtained for the nine mcr-1-negative CSTS isolates which had inhibition zone diameter higher than 17 mm (Table S1)*.

**Table 3:** Virulence markers detected in the 79 colistin-resistant *E. coli* isolates.

**Virulence marker**		**Number of positive isolates**	**% of positive isolates**
O antigens	O141	21	27
	O149	12	15
	O139	8	10
	O8(abc)	3	4
F antigens	F18	29	37
	F4	27	34
	F5	0	0
	F41	0	0
Toxins	*hlyA*	61	77
	*estII*	44	56
	*astA*	41	52
	*elt*	32	40
	*estI*	31	39
	*stx_2*e*_*	27	34
	*ehxA*	0	0
Adhesins	*iha*	37	47
	*orfA*	30	38
	*orfB*	29	37
	*paa*	27	34
	*lpfA*-O113	17	22
	*eae*	1	1
	*lpfA*-O157	0	0
	*saa*	0	0
	*efa1*	0	0
	*bfpA*	0	0
	*aggR*	0	0
Iron acquisition	*fyuA*	8	10
	*irp2*	8	10
Others	*terE*	67	85
	*ecs1763*	48	61
	*ureD*	37	47
	*epeA*	0	0
	*ecf1*	0	0
	*nleB*	0	0
	*ipaH*	0	0

**Table 4:** Distribution of virulence markers, antimicrobial resistance profiles, and genetic profiles of resistance (GPR) among the 81 colistin-resistant or *mcr-1*-positive *E. coli* isolates.

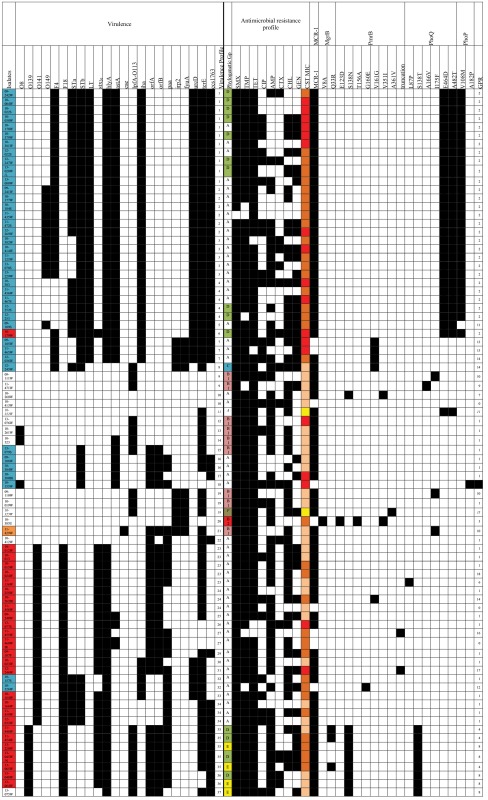

*Isolates with code ending in “S” were isolated from suckling piglets, “F” from fattening pigs, “W” for post-weaning piglets, “U” from urine, “Bl” from cases of septicemia, “N” from nervous system,” L” from lymphatic node. No data related to the status of the animals were available for the other isolates. First column: STEC (red), EPEC (orange), ETEC (blue). For virulence marker ou genetic modifications, black cells indicate the presence of the virulence marker, gene or mutation*.

*Phylogenetic groups: A (white cells), B1 (pink cells), B2 (red cells), C (blue cells), D (green cells), E (yellow cells) and F (brown cells)*.

*SMX, Sulfamethoxazole, TMP, Trimethoprim, TET, Tetracycline, CIP, Ciprofloxacin, AMP, Ampicillin, CTX, Cefotaxime, CHL, Chloramphenicol, GEN, gentamicin; MIC of colistin in mg/L: 2 (yellow), 4 (orange pale), 8 (dark orange), 16 (red)*.

*Genetic profiles of resistance (GPR) correspond to the combination of genetic characteristics of columns MCR-1 to A182P. The impact on colistin resistance of the indicated missense mutations is not demonstrated*.

*The PmrA sequence could not be determined for the 10-024 isolate*.

*The mcr-1-positive CSTS isolates 10-322 and 10-325 are in italics*.

The *mgrB* gene, which encodes a short 47-amino-acid transmembrane protein, negatively regulating the histidine-kinase of PhoQ in the PhoP/PhoQ two-component system (Lippa and Goulian, 2012), was sequenced in the 79 CSTR isolates and in the 11 *mcr-1*-positive or -negative CSTS isolates. Two mutations V8A (one *mcr-1* negative isolate) and Q33R (three *mcr-1*-positive isolates) were detected only in CSTR isolates. Similarly, in *Klebsiella pneumoniae*, mutation L8Y in the MgrB sequence has already been reported in CSTR isolates and the D31N mutation is deleterious, affecting protein function (Olaitan et al., 2014a). Premature stop codons and different insertion sequences (IS) in the *mgrB* gene, its promotor or the flanking regions have also been observed in *in vitro* CSTR *Klebsiella* mutants or in clinical isolates (Olaitan et al., 2014a).

The sequences obtained for PhoP lead to a protein of 233 amino acids for all isolates. Missense mutations observed only in CSTR isolates were detected in two positions, including V108M detected in 28 CSTR *mcr-1*-positive isolates, and A182P detected in a single CSTR *mcr-1*-negative isolate. The V108M mutation seems frequent in *E. coli* according to the sequences available in GenBank, but its impact on CST resistance is unknown. Conversely, to our knowledge, the A182P sequence modification in the DNA binding region of PhoP has never been reported before and its impact on colistin resistance is unknown. Other modifications of the PhoP sequence have been described such as L26Q in CSTR *K. pneumoniae* (Olaitan et al., 2014a).

Regarding PhoQ, results showed that the DNA sequences coded for a protein of 486 amino acids for all isolates. Several mutations were detected, all in *mcr-1*-positive isolates: L87P (1 CSTR isolate), S138T (8 CSTR isolates), A166V (1 CSTR isolate), I175F (3 CSTR isolates), and E464D and A482T (both in the same 30 CSTS or CSTR isolates). To our knowledge, the PhoQ mutations detected in this study have not yet been described in *E. coli* and further studies are needed to evaluate their impact on polymyxin resistance. Other modifications, sometimes similar to the ones that we observed, have been reported in *E. coli* (A71S, H102Q, V103I,−139R, E155K, L184F, I338V, G413W, L433F) (Anjum et al., 2016), CSTR *K. pneumoniae* (L96P, S174N, L348Q, and G385S) (Olaitan et al., 2014b), or in *Pseudomonas aeruginosa* (Q133E) (Choi and Ko, 2014). Minagawa et al. (2005) showed that D179, near to the I175F mutation observed here, plays an essential role in signal transfer between the Mg^2+^-sensory and kinase domains of PhoQ.

The PmrA sequence could not be determined for the 10-024 *mcr-1*-positive isolate. For the other isolates, the 222 amino-acids PmrA sequences were identical, except for the CSTR isolate 11-455 which had a G144S substitution, previously observed in a CSTS *E. coli* strain (Quesada et al., 2015). Substitutions resulting in constitutive activation of this two-component system in *E. coli* (V89I, A111S, A115G, A122G) (Anjum et al., 2016), *Salmonella enterica* (G15R, G53E, G53R, R81H, R81C), *K. pneumoniae* (S42N, G53C, G53S), or *Enterobacter aerogenes* (G53C) have already been described (Poirel et al., 2017).

For the 363-amino-acid PmrB protein, sequences displayed various polymorphisms, the following being detected only in resistant isolates: E123D (one *mcr-1*-negative isolate), S138N (9 *mcr-1*-positive isolates), T156A (one *mcr-1*-negative isolate), G160E *(*one *mcr-1*-negative isolate), V161G (in two *mcr-1*-negative and three *mcr-1*-positive isolates) andV351I (one *mcr-1*-negative and one *mcr-1*-positive isolates). The substitution A361V was detected only in the *mcr-1*-positive CSTS isolate 10-325. A D283G mutation previously observed by Quesada et al. (2015) in CSTS *E. coli* was also detected in *mcr-1*-positive and *mcr-1*-negative isolates. For the *mcr-1*-negative isolate 11-455, due to a nucleotide deletion, a frameshift was observed after the 149th amino acid, resulting in a truncated protein. For the *mcr-1*-positive isolate 12-246, a deletion of five nucleotides resulted in a truncated protein containing only the first five amino acids of PmrB. Finally, an insertion of five amino acids (KDQYK) was detected at position 357 of PmrB in the CSTS 09-189 isolate. Previous authors (Olaitan et al., 2014b; Anjum et al., 2016) have described mutations in PmrB (M363T in *E. coli*, E121A, S124P, V161M, V161L, or V161G in CSTR *S. enterica*, and T140P and T157P in CSTR *K. pneumoniae*) at positions close to the ones that we report in this study. The V161G mutation has also been detected in the kinase domain of PmrB in a CSTR *E. coli* (Quesada et al., 2015). However, not all missense mutations in *pmrA/pmrB* result in colistin resistance (Olaitan et al., 2014b), and the impact of the mutations detected in this study needs to be experimentally demonstrated.

The mutations that were detected in the 81 CSTR or *mcr-1*-positive isolates are reported in Tables 2, 4. The combinations of modifications led to 19 different genetic profiles of resistance (GPR), encoded GPR 0 to 18, for the CSTR or *mcr-1*-positive isolates. For three CSTR isolates (UB-10-413, UB-11-456, and 11-460 with CST MICs of 4 or 8 mg/L, GPR 0), no mutation was recorded and they did not carry the *mcr-1* gene. For these CSTR isolates, other modifications of the genome, such as in the *arnBCADTEF* operon, or the *pmrE, pmrC, eptB, mgrR*, or *lpxM* genes (Olaitan et al., 2014b) may explain resistance to CST, but no further characterization of these isolates has been attempted yet.

Six other CSTR isolates did not carry the *mcr-1* gene and showed five different GPR, including mutations (G160E, V161G, E123D, T156A, V351I) in or truncation of PmrB, alone or with MgrB modifications, such as isolate 10-385 harboring one mutation in MgrB (V8A) and three mutations in PmrB. One isolate, UB-10-155, had only one PhoP modification. In particular, the two isolates harboring only the V161G mutation in PmrB had a MIC of 16 mg/L.

Five isolates harbored the *mcr-1* gene and presented the only two polymorphisms: S138N in PmrB and S138T in PhoQ. Their CST MICs were 4 or 8 mg/L. Three of these isolates belonged to the E phylogroup. The fourth isolate of this phylogroup harbored the same mutations and the other mutation (Q33R) was present in MgrB. All four isolates of this phylogroup were collected in 2013.

Twenty-two isolates had the *mcr-1* gene and no other modification was detected in the sequenced genes (GPR 1). Their CST MICs were 4, 8, or 16 mg/L and they were collected between 2009 and 2013. They belonged to phylogroups A (17 isolates) and B1 (5 isolates).

Twenty-eight isolates harbored the *mcr-1* gene and two mutations in PhoQ and V108M in PhoP (GPR 2). Their MICs were 8 or 16 mg/L and they belonged to phylogroups A (14 isolates) and D (9 isolates).

### Virulence markers

The high-throughput real-time PCR micro-array confirmed that all isolates were positive for the *E. coli* control *wecA* gene. The genes detected in the 79 CSTR isolates are given in Table 3.

Regarding the O antigens, O141 (27%) was found more frequently than O149 (15%) and O139 (10%) in the CSTR collection. The O141 and O139 percentages seemed higher than reported by others (Frydendahl, 2002; Chen et al., 2004; Chapman et al., 2006; Smith et al., 2010). The determinant *lpfA*_O157_, rarely found in other studies (Frydendahl, 2002; Chen et al., 2004; Byun et al., 2013; Baranzoni et al., 2016), was not detected.

The F18 and F4 fimbrial genes were detected in approximately one-third of CSTR isolates. F5 and F41 antigens were not detected.

When cultured on Mueller-Hinton medium containing sheep blood, 61 isolates (77%) produced a hemolysis zone, consistent with the high prevalence of the alpha-hemolysin gene *hlyA* (77% of isolates), being present in all hemolytic isolates and only in hemolytic isolates. According to Chapman et al. (2006), the *hlyA* gene is found significantly more frequently in strains from weaners (72%) than in strains from scouring neonatal pigs (10%) or commensal strains (4%).

A total of 39 isolates had the ST or LT-encoding genes and could be classified as ETEC. More than half of the CSTR isolates contained the toxin genes *estII* (56%) or *astA* (52%). The percentages in the literature for the STb determinant vary from 25 to 31% of neonatal strains (Chapman et al., 2006; Byun et al., 2013) to more than 80% of strains from PWD (Chapman et al., 2006; Smith et al., 2010). The *astA* gene is also frequently reported, being present in 40% to 50% of STEC, neonatal diarrhea, and PWD strains (Osek, 2003; Byun et al., 2013; Baranzoni et al., 2016).

Genes coding for STa and LT toxins were present in 39–40% of our CSTR isolates, whereas previously published percentages for STa vary from 24% in strains of neonatal pigs (Byun et al., 2013) to 92% in O141 strains from PWD pigs (Smith et al., 2010), and the LT determinant ranges from 15% in neonatal strains (Chapman et al., 2006) to 76% for O149 strains (Smith et al., 2010). According to Fairbrother et al., the gene encoding STb is present on a plasmid encoding LT in LT^+^ F4^+^ ETEC (Fairbrother et al., 2005): all 32 CSTR isolates positive for LT were also positive for STb.

One-third of our CSTR isolates harbored the *stx*_2*e*_ gene (27 isolates, 34%), and could thus be classified as STEC. All these isolates tested positive for the F18 target.

The toxin gene *ehxA* was not detected in our CSTR isolates, whereas it has been reported in STEC from finishing pigs (Baranzoni et al., 2016). The genes *saa, efa1, bfpA*, and *aggR* were also absent in our CSTR isolates, the first three genes also lacking in the 181 STEC isolated from healthy finishing pigs in the USA (Baranzoni et al., 2016).

The adhesin genes *iha, orfA, orfB*, and *paa* were found in one-third of the isolates or more. Very high percentages of *iha*-positive isolates have been described by different authors, particularly for STEC, O149, or O141 strains (Wu et al., 2007; Smith et al., 2010; Tseng et al., 2014). Chapman et al. observed that the *orfA* and *orfB* determinants were more frequent in strains from weaners than in strains from neonatal piglets (Chapman et al., 2006). The *paa* gene was present in 0% of cases of PWD in Australia (Smith et al., 2010), but in more than 80% STEC isolates from finishing pigs (Tseng et al., 2014). The *lpf*_O113_ was present in only 22% of CSTR isolates, compared with 85% positive STEC from finishing pigs (Tseng et al., 2014).

Only one isolates (11-429) was positive for the *eae* intimin gene; this isolate also possessed the o*rfA, orfB, paa, fyuA, irp2*, and *terE* genes, but lacked the *stx*_2*e*_ gene, and was thus an EPEC. No isolate was a typical EHEC (Bugarel et al., 2011).

The *irp*2 and *fyuA* genes, both belonging to the high pathogenicity island, were present in a limited number of isolates (10%). Conversely, *terE* conferring tellurite resistance was the most prevalent marker (85% of isolates), and was more frequent in our collection than others (17%) (Baranzoni et al., 2016).

A total of 37 different combinations of virulence genes was observed from the 79 CSTR and the 2 *mcr-1*-positive CSTS studied isolates (Table 4). Only two CSTR isolates (10-260 and 10-413) had no virulence markers. Seventeen CSTR isolates (21% of the CSTR isolates) had a total of 11 markers, and 24 CSTR isolates (30% of the CSTR isolates) had 10 markers. Twenty-one CSTR isolates (27% of the CSTR isolates) had a unique virulence profile (VP).

The most frequent combination of virulence markers (VP1: F4, *estI, estII, elt, hlyA, astA, iha, ureD, terE*, and *ecs1763*) was shared by 11 CSTR isolates from phylogroups A or D, collected between 2009 and 2013. All had the *mcr-1* gene and the same three mutations in PhoP and PhoQ (GPR 2). Two of these isolates were CTX-resistant. Six other CSTR isolates had the same virulence markers; additionally they belonged to the O149 serogroup (VP2) and all were from phylogroup A and had the same GPR 2. Moreover, all 12 O149-positive isolates (VP2, VP3, or VP5) harbored the genes coding for the toxins STb, LT, alpha-hemolysin, and EAST1, the adhesins *iha*, and the markers *ureD, terE*, and *esc1763*. All belonged to phylogroup A and had the GPR 2. These results are consistent with the various associations of F4, O149, LT, STa, STb, EAST-1, and alpha-hemolysin previously classified as important ETEC pathotypes for piglets (Fairbrother and Gyles, 2012).

The eight CSTR O139 isolates (VP35, VP36, or VP37) carried the F18, *hlyA, lpfA*-O113, *orfA, orfB*, and *ecs1763* genes, and seven of them also harbored the *stx*_2*e*_, and *paa* genes. They belonged to the phylogroups D or E. They were characterized by the GPR 4 or 8. Importantly three of these isolates were CTX-resistant. Fairbrother and Gyles reported that F18, AIDA, Stx2e, alpha-hemolysin, (and also EAST-1, not detected in our O139 CSTR isolates) are common STEC pathotypes (Fairbrother and Gyles, 2012).

Among the 21 CSTR O141 isolates, all belonging to the phylogroup A, all had the F18 and the *hlyA* genes, and 19 of them the *stx*_2*e*_ gene, 15 had the *orfA* and *orfB* genes. Eighteen possessed the *mcr-1* gene and two had been isolated from septicemia (10-262 and 11-460). Again, such combinations of virulence genes have already been reported (Fairbrother and Gyles, 2012) for strains isolated from ED or PWD.

This study is an unprecedented phenotypic and genetic characterization of more than 80 CSTR or *mcr-1*-positive CSTS *E. coli* isolates obtained from diseased pigs in France between 2009 and 2013. In the Kusumoto et al. study (Kusumoto et al., 2016) on pig pathogenic strains in Japan, there was a high proportion (45%) of CSTR *E. coli*, contrary to the low proportion in healthy animals; the presence of the *mcr-1* gene in isolates belonging to the four main pathogenic serotypes suggested that plasmid-mediated horizontal transfer, and not dissemination of a specific clone, was responsible for the spread of *mcr-1*-positive isolates. In our study, we revealed that, although most isolates had a unique PFGE profile, several particular combinations of virulence genes, phylogenetic groups, and GPR were observed on a number of occasions. In particular, the GPR 2, including the *mcr-1* gene and three mutations in PhoP and PhoQ genes was detected in 28 isolates with five closely related VP and obtained from post-weaning or suckling piglets between 2009 and 2013. Lacking precise epidemiological data on the farms from which these isolates were obtained, it is difficult to determine whether their presence in different farms results from close geographical position, transfer of animals, or any other reason. The persistence over several years of such isolates in pig farms may also be related to the administration of CST or of other antibiotics, given the presence of genes coding for resistance to antimicrobials other than CST on the *mcr-1* conjugative plasmids. This persistence may also be linked to a low fitness cost of *mcr-1*-harboring plasmids as described for IncX3 plasmids (Kong et al., 2017). Conversely, 22 unique VP, as well as five unique GPR were encountered, underlining the overall diversity of our collection, and the probable transfer of *mcr-1* plasmids between different isolates. Although we detected the *mcr-1* gene in the majority (89%) of the CSTR isolates, nine of them did not contain this gene, nor the *mcr-2* and *mcr-3* genes. For three of these isolates which had three different VPs, we did not detect mutations in the MgrB, PhoP, PhoQ, PmrA, or PmrB genes, and further investigation is now needed to identify their CST-resistance mechanisms and evaluate the impact on polymyxin resistance of the mutations detected in the other isolates. The characterization of the *mcr-1* plasmids will also be undertaken to evaluate their diversity and dissemination. Finally the presence of the *mcr-1* gene in multidrug-resistant ETEC and STEC strains is worrisome for animal and human health.

In conclusion, it is of major importance to further monitor and characterize CST resistance in pigs and other livestock production systems. Improvement in biosecurity, breeding conditions and alternative strategies (e.g., vaccination) hold promise for the effort to reduce the use of CST and other antimicrobials in livestock animals.

## Author contributions

SD, EJ, IK, and PF: Contributed to the design of the work, contributed to the acquisition, analysis and the interpretation of the data, wrote the paper; LL and DD: contributed to the design of the work, contributed to the acquisition, analysis, and the interpretation of the data, final approval of the submitted version.

### Conflict of interest statement

The authors declare that the research was conducted in the absence of any commercial or financial relationships that could be construed as a potential conflict of interest.

## Supplementary material

Figure S1 PFGE profiles of the colistin-resistant *E. coli* isolates.

Table S1 Primers used for sequencing PmrA, PmrB, mgrB, PhoP, PhoQ and mcr-1.

Table S2 Disk diffusion, colistin MICs and presence of the mcr-1 gene in the isolates.
