# Supplementary figures and images for: Characterization of Colistin-Resistant Escherichia coli Isolated from Diseased Pigs in France
Source: Front Microbiol. 2017 Nov 21;8:2278. doi: 10.3389/fmicb.2017.02278 (PMC5702452; doi:10.3389/fmicb.2017.02278)

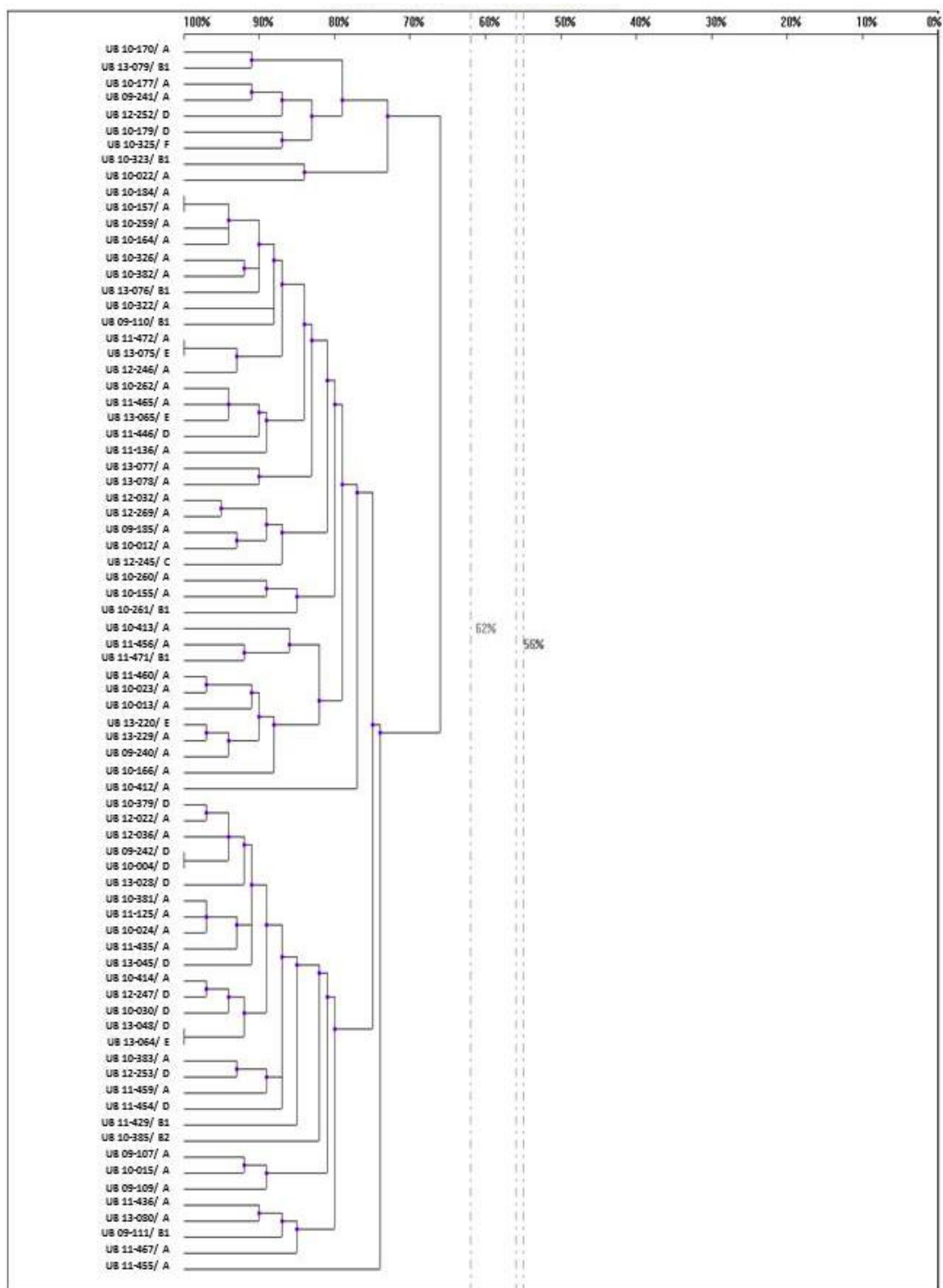

Supplement: Figure S1 — PFGE profiles of the colistin-resistant E. coli isolates. [file Image1.PDF]
