# Supplementary material for: Characterization of Colistin-Resistant Escherichia coli Isolated from Diseased Pigs in France
Source: Front Microbiol. 2017 Nov 21;8:2278. doi: 10.3389/fmicb.2017.02278 (PMC5702452; doi:10.3389/fmicb.2017.02278)
Supplement: Table S1 — Primers used for sequencing PmrA, PmrB, mgrB, PhoP, PhoQ and mcr-1. [file Table1.pdf]

Table S1: Primers used for sequencing pmrA, pmrB, mgrB, phoP, phoQ and mcr-1

PmrAB	CS2-PM290-1-rev	TACGGTAGCAGAGACTTGGTCTGCGGGCGGTGAGAATTAAC
PmrAB	CS2-PM290R1-2-rev	TACGGTAGCAGAGACTTGGTCTGTGAGGATCAGTACCGGCA
PmrAB	CS2-PM290R2-3-rev	TACGGTAGCAGAGACTTGGTCTGATCAGTACTGGCAGGGTGT
PmrAB	CS2-PM383R1-4-rev	TACGGTAGCAGAGACTTGGTCTCCCTGATTATTATGGCGTCGT
PmrAB	CS2-PM383R2-5-rev	TACGGTAGCAGAGACTTGGTCTGCCCTGATTATTATGGCGTCG
PmrAB	CS2-PM445R1-6-rev	TACGGTAGCAGAGACTTGGTCTGCGACCCATATTCAGCGTCA
PmrAB	CS2-PM569-7-rev	TACGGTAGCAGAGACTTGGTCTGAGAATTTCCCGATGCACCG
PmrAB	CS2-PM648-8-rev	TACGGTAGCAGAGACTTGGTCTCTTTGTCGCGCAGGTTATGG
PmrAB	CS2-PM648R1-9-rev	TACGGTAGCAGAGACTTGGTCTCTTTGTCGCGCAGATTGTGG
PmrAB	CS2-PM807-10-rev	TACGGTAGCAGAGACTTGGTCTTCAGCTCGAATACCAGCAGAA
PmrAB	CS2-PM807R2-11-rev	TACGGTAGCAGAGACTTGGTCTGCCATAGCCAGAAGACGCT
PmrAB	CS2-PM903-12-rev	TACGGTAGCAGAGACTTGGTCTGCATGATGTGGCGATCGTTG
PmrAB	CS2-PM959-13-rev	TACGGTAGCAGAGACTTGGTCTGCTAACCATAAAGACGCCGG
PmrAB	CS2-PM959R1-14-rev	TACGGTAGCAGAGACTTGGTCTTAAAGACGCCGGGGACAATC
PmrAB	CS2-PM959R2-15-rev	TACGGTAGCAGAGACTTGGTCTTGACCATAAAGACGCCGGG
PmrAB	CS2-PM1091-16-rev	TACGGTAGCAGAGACTTGGTCTGATTTCGAGGGTGGCGCTAT
PmrAB	CS2-PM1091R1-17-rev	TACGGTAGCAGAGACTTGGTCTCGCTGTGAATGGCAATGGG
PmrAB	CS2-PM1157-18-rev	TACGGTAGCAGAGACTTGGTCTCCTTTCGTTATCCAGCGTGC
PmrAB	CS2-PM1157R1-19-rev	TACGGTAGCAGAGACTTGGTCTTTCATTATCGAGCGTGCTGGT
PmrAB	CS2-PM1227-20-rev	TACGGTAGCAGAGACTTGGTCTGCAGTTCCAGATGCAAACGC
PmrAB	CS2-PM1340-21-rev	TACGGTAGCAGAGACTTGGTCTGGAAGAAAATGACTGTCCGGC
PmrAB	CS2-PM1340R1-22-rev	TACGGTAGCAGAGACTTGGTCTAAGAAAATGACTGTCCGGCAC
PmrAB	CS2-PM1496-23-rev	TACGGTAGCAGAGACTTGGTCTTAACATCCGCAGCAGGGTTG
PmrAB	CS2-PM1575-24-rev	TACGGTAGCAGAGACTTGGTCTCGCCATCTTCTTGCAGCTTAA
PmrAB	CS2-PM1719-25-rev	TACGGTAGCAGAGACTTGGTCTGCAACTGTGTAATGCGGCTG
PmrAB	CS2-PM1854-26-rev	TACGGTAGCAGAGACTTGGTCTCTACCGTGTTCAGCGTGCT
PmrAB	CS2-PM1902-27-rev	TACGGTAGCAGAGACTTGGTCTGGTTAACGGAGGAGAGTGCA
MgrB	CS2-Mg216-28-rev	TACGGTAGCAGAGACTTGGTCTGCTTTGGGCGCAGGTATTC
MgrB	CS2-Mg376-29-rev	TACGGTAGCAGAGACTTGGTCTGAAGCAAGCGACTCATTCCG
MgrB	CS2-Mg504-30-rev	TACGGTAGCAGAGACTTGGTCTAAAACGCCATATCCGCTGAGT
MgrB	CS2-Mg630-31-rev	TACGGTAGCAGAGACTTGGTCTAGCGGGCAATCTGTTATCCC
PhoP	CS2-PhP165-32-rev	TACGGTAGCAGAGACTTGGTCTATTCAGGCACAATATCCGCAAG
PhoP	CS2-PhP400-33-rev	TACGGTAGCAGAGACTTGGTCTGGCTTCACAGGTCATTTCGC
PhoP	CS2-PhP615-34-rev	TACGGTAGCAGAGACTTGGTCTGATCTCGGATTGCCTGACGA
PhoP	CS2-PhP824-35-rev	TACGGTAGCAGAGACTTGGTCTCCCATAACCACATAATCGCGT
PhoP	CS2-PhP1012-36-rev	TACGGTAGCAGAGACTTGGTCTAGAGAAAGCCCGCCTGAAAG
PhoP	CS2-PhP1168-37-rev	TACGGTAGCAGAGACTTGGTCTTCACAACTGGGAAGTGCTGG
PhoP	CS2-PhP1243-38-rev	TACGGTAGCAGAGACTTGGTCTTTGCGTATCAATCCACCCTGA
PhoP	CS2-PhP1377-39-rev	TACGGTAGCAGAGACTTGGTCTCGAAGGGAATCTGGGCCTTT
PhoP	CS2-PhP1421-40-rev	TACGGTAGCAGAGACTTGGTCTCTTCCACCATGCCGCATAAAG
PhoP	CS2-PhP1581-41-rev	TACGGTAGCAGAGACTTGGTCTAACCCGTACACCACCCAGAT
PhoP	CS2-PhP1615-42-rev	TACGGTAGCAGAGACTTGGTCTAAGAGTTCGTCACCTCGCTG
PhoQ	CS2-PhQ250-43-rev	TACGGTAGCAGAGACTTGGTCTCTGGGCAAGGTGTAGGGC

PhoQ	CS2-PhQ456-44-rev	TACGGTAGCAGAGACTTGGTCTCTTTGTCGGTGAGCAGAACG
PhoQ	CS2-PhQ434-45-rev	TACGGTAGCAGAGACTTGGTCTTTTGTCGAAGTGATGGGCAAC
PhoQ	CS2-PhQ538-46-rev	TACGGTAGCAGAGACTTGGTCTTGGACAATCTCACCTCAGCG
PhoQ	CS2-PhQ754-47-rev	TACGGTAGCAGAGACTTGGTCTTCACCGACCTGACCCATAGT
PhoQ	CS2-PhQ920-48-rev	TACGGTAGCAGAGACTTGGTCTAGTTTACGCCCCATCGAAGC
PhoQ	CS2-PhQ1139-49-rev	TACGGTAGCAGAGACTTGGTCTCAGGAAGTGCGGGAAGATGA
PhoQ	CS2-PhQ1339-50-rev	TACGGTAGCAGAGACTTGGTCTATATCGACAAGCAAAGCCCCA
PhoQ	CS2-PhQ1528-51-rev	TACGGTAGCAGAGACTTGGTCTGGGTACGTTTTCTGTTGGCAA
PhoQ	CS2-PhQ1640-52-rev	TACGGTAGCAGAGACTTGGTCTATTCAGGCACAATATCCGCAAG
PhoQ	CS2-PhQ201-53-rev	TACGGTAGCAGAGACTTGGTCTTGAGGGTAAAATCGTCGCCG
PhoQ	CS2-PhQ337-54-rev	TACGGTAGCAGAGACTTGGTCTTTGTGGTCGAGGATGATGGC
mcr-1	CS2-mcr357-55-rev	TACGGTAGCAGAGACTTGGTCTACAGCGTGGTGATCAGTAGC
mcr-2	CS2-mcr417-56-rev	TACGGTAGCAGAGACTTGGTCTCCGCGCCCATGATTAATAGC
mcr-3	CS2-mcr511-57-rev	TACGGTAGCAGAGACTTGGTCTAGATCCTTGGTCTCGGCTTG
mcr-4	CS2-mcr669-58-rev	TACGGTAGCAGAGACTTGGTCTCCACAGGCAGTAAAATCAGCG
mcr-5	CS2-mcr769-59-rev	TACGGTAGCAGAGACTTGGTCTGCAAGCTTACCCACCGAGTA
mcr-6	CS2-mcr856-60-rev	TACGGTAGCAGAGACTTGGTCTCGCATATCAGGCTTGGTTGC
mcr-7	CS2-mcr964-61-rev	TACGGTAGCAGAGACTTGGTCTCCATCGATCTTGGCAAGCTG
mcr-8	CS2-mcr1102-62-rev	TACGGTAGCAGAGACTTGGTCTTCCAGCGTATCCAGCACATTT
mcr-9	CS2-mcr1296-63-rev	TACGGTAGCAGAGACTTGGTCTCGTTATTGGCAGCGACAAAGT
mcr-10	CS2-mcr1403-64-rev	TACGGTAGCAGAGACTTGGTCTATTACCTTCACACACTGGCGT
mcr-11	CS2-mcr1475-65-rev	TACGGTAGCAGAGACTTGGTCTGAAATCATCGGTGGCAAGCAA
mcr-12	CS2-mcr1661-66-rev	TACGGTAGCAGAGACTTGGTCTGCCAGTTTGCTTATCCGTCC
mcr-13	CS2-mcr1823-67-rev	TACGGTAGCAGAGACTTGGTCTTACGAATGGAGTGTGCGGTG
mcr-14	CS2-mcr1991-68-rev	TACGGTAGCAGAGACTTGGTCTTCCAATGCCCCATACCCAGA
PmrAB	CS1-PM27-1-for	ACACTGACGACATGGTTCTACAAACTTGCAGGAGAGTGAGTGAA
PmrAB	CS1-PM164-2-for	ACACTGACGACATGGTTCTACACCTTGAGGCCGGTCATTACA
PmrAB	CS1-PM208-3-for	ACACTGACGACATGGTTCTACATACCCGATGAAGATGGGCTG
PmrAB	CS1-PM208R1-4-for	ACACTGACGACATGGTTCTACATACCCGACGAAGATGGACTG
PmrAB	CS1-PM288-5-for	ACACTGACGACATGGTTCTACACGCGATACCCTTACCGACAA
PmrAB	CS1-PM288R1-6-for	ACACTGACGACATGGTTCTACAGCCGACGACTATCTGGTGAA
PmrAB	CS1-PM388-7-for	ACACTGACGACATGGTTCTACACCCTGCTACGACGCCATAAT
PmrAB	CS1-PM550-8-for	ACACTGACGACATGGTTCTACACGGTGCATCGGGAAATTCTC
PmrAB	CS1-PM550R1-9-for	ACACTGACGACATGGTTCTACAATGAACCCTCGACCAACACC
PmrAB	CS1-PM629-10-for	ACACTGACGACATGGTTCTACACCATAACCTGCGCGACAAAG
PmrAB	CS1-PM629R1-11-for	ACACTGACGACATGGTTCTACACAATCTGCGCGACAAAGTGG
PmrAB	CS1-PM737-12-for	ACACTGACGACATGGTTCTACAACGCCAACCAATATCACTACGT
PmrAB	CS1-PM737R1-13-for	ACACTGACGACATGGTTCTACAGTGCGCGGATTTGGCTATATG
PmrAB	CS1-PM737R2-14-for	ACACTGACGACATGGTTCTACAGTTTTCTGCGCCGACCAATAT
PmrAB	CS1-PM826-15-for	ACACTGACGACATGGTTCTACAGGCATGAAAGTACCGAGCAG
PmrAB	CS1-PM932-16-for	ACACTGACGACATGGTTCTACAGATTGTCCCCGGCGTCTTTA
PmrAB	CS1-PM941-17-for	ACACTGACGACATGGTTCTACACGGCGTCTTTATGGTTAGCC
PmrAB	CS1-PM1068-18-for	ACACTGACGACATGGTTCTACAATTCATAGCGCCACCCTCG
PmrAB	CS1-PM1068R2-19-for	ACACTGACGACATGGTTCTACACCCATTGCCATTCACAGCG
PmrAB	CS1-PM1072-20-for	ACACTGACGACATGGTTCTACAATAGCGCCACCCTCGAAATC

PmrAB	CS1-PM1215-21-for	ACACTGACGACATGGTTCTACACATCTGGAACTGCTGGCGAA
PmrAB	CS1-PM1220-22-for	ACACTGACGACATGGTTCTACAGGAACTGCTGGCGAAAACG
PmrAB	CS1-PM1317-23-for	ACACTGACGACATGGTTCTACACGTGCCGGACAGTCATTTTC
PmrAB	CS1-PM1481-24-for	ACACTGACGACATGGTTCTACACCTGCTGCGGATGTTATTGC
PmrAB	CS1-PM1580-25-for	ACACTGACGACATGGTTCTACAGGTCATGGCCGTTGAAGATG
PmrAB	CS1-PM1628-26-for	ACACTGACGACATGGTTCTACACGGGGAGTTGAGTAAAGCGT
PmrAB	CS1-PM1665-27-for	ACACTGACGACATGGTTCTACACGTTATGGCGGGATTGGTCT
MgrB	CS1-Mg-39-28-for	ACACTGACGACATGGTTCTACAAAACCACAAGATGAGCCCGAT
MgrB	CS1-Mg126-29-for	ACACTGACGACATGGTTCTACATCACCACGGGATAAACTGGTT
MgrB	CS1-Mg236-30-for	ACACTGACGACATGGTTCTACACGACAACCAGAACGACCCA
MgrB	CS1-Mg357-31-for	ACACTGACGACATGGTTCTACACGGAATGAGTCGCTTGCTTC
PhoP	CS1-PhP-74-32-for	ACACTGACGACATGGTTCTACAGCTTTCGCCACGTAACAGC
PhoP	CS1-PhP139-33-for	ACACTGACGACATGGTTCTACACACTTCTTGCGGATATTGTGCC
PhoP	CS1-PhP347-34-for	ACACTGACGACATGGTTCTACATCACGGCGAGAGAGATCAAC
PhoP	CS1-PhP574-35-for	ACACTGACGACATGGTTCTACAGCGAATCAGTGACAGACCGT
PhoP	CS1-PhP744-36-for	ACACTGACGACATGGTTCTACACTTCAACAACCAGTACGCGC
PhoP	CS1-PhP891-37-for	ACACTGACGACATGGTTCTACATAAACAGGCCGGACAGCATC
PhoP	CS1-PhP999-38-for	ACACTGACGACATGGTTCTACAGGCGGGCTTTCTCTTCTTCT
PhoP	CS1-PhP1120-39-for	ACACTGACGACATGGTTCTACAGCGACGCATAACTGTCTGGA
PhoP	CS1-PhP1151-40-for	ACACTGACGACATGGTTCTACACCAGCACTTCCCAGTTGTGA
PhoP	CS1-PhP1334-41-for	ACACTGACGACATGGTTCTACACCAGATGCTGCAATACCGC
PhoP	CS1-PhP1358-42-for	ACACTGACGACATGGTTCTACAAAAGGCCCAGGTTCCCTTC
PhoP	CS1-PhP1359-43-for	ACACTGACGACATGGTTCTACAAAGCCCCAGGTTTCCTTCG
PhoQ	CS1-PhQ1-44-for	ACACTGACGACATGGTTCTACATCGGGCCAGTTAAGAGTGAG
PhoQ	CS1-PhQ200-45-for	ACACTGACGACATGGTTCTACAATATTGCTCGGTGATTTCGCG
PhoQ	CS1-PhQ310-46-for	ACACTGACGACATGGTTCTACAATACCGGGGCCATCATCCT
PhoQ	CS1-PhQ488-47-for	ACACTGACGACATGGTTCTACATGACCCCTTTGCGTTGATACA
PhoQ	CS1-PhQ648-48-for	ACACTGACGACATGGTTCTACACATTACCGGCTCAGCATCAC
PhoQ	CS1-PhQ881-49-for	ACACTGACGACATGGTTCTACACGCGGACTTCTTTTGCCAG
PhoQ	CS1-PhQ1066-50-for	ACACTGACGACATGGTTCTACAGATGTTGCCGGGTAGACGT
PhoQ	CS1-PhQ1270-51-for	ACACTGACGACATGGTTCTACACGTTGCGCCCATAAAAGCTG
PhoQ	CS1-PhQ1407-52-for	ACACTGACGACATGGTTCTACAGCCACGTAACAGCCGAAAC
PhoQ	CS1-PhQ-68-53-for	ACACTGACGACATGGTTCTACAGGAAAGATGACCCGCCTGTA
PhoQ	CS1-PhQ109-54-for	ACACTGACGACATGGTTCTACATTCATCTTTCGGCGCAGAATG
mcr-1	CS1-mcr110-55-for	ACACTGACGACATGGTTCTACAATTGCCGTAATTATCCCACCG
mcr-1	CS1-mcr195-56-for	ACACTGACGACATGGTTCTACACCGTTTGTTCTTGTGGCGAG
mcr-1	CS1-mcr267-57-for	ACACTGACGACATGGTTCTACAAGCCAAACCTATCCCATCGC
mcr-1	CS1-mcr398-58-for	ACACTGACGACATGGTTCTACAGCTATTAATCATGGGCGCGG
mcr-1	CS1-mcr492-59-for	ACACTGACGACATGGTTCTACACAAGCCGAGACCAAGGATCT
mcr-1	CS1-mcr608-60-for	ACACTGACGACATGGTTCTACAGGGTTTGATGCGCCGATTG
mcr-1	CS1-mcr741-61-for	ACACTGACGACATGGTTCTACAATGCCAATCTACTCGGTGGG
mcr-1	CS1-mcr837-62-for	ACACTGACGACATGGTTCTACAGCAACCAAGCCTGATATGCG
mcr-1	CS1-mcr1041-63-for	ACACTGACGACATGGTTCTACAGGCGCGGATGAGTATGATGT
mcr-1	CS1-mcr1141-64-for	ACACTGACGACATGGTTCTACACGGACTCAAAAGGCGTGATG
mcr-1	CS1-mcr1251-65-for	ACACTGACGACATGGTTCTACAGTCGGTATGCTCGTTGGCTT

mcr-1	CS1-mcr1411-66-for	ACACTGACGACATGGTTCTACACCAAGTGCGAACATCAGTCC
mcr-1	CS1-mcr1559-67-for	ACACTGACGACATGGTTCTACATCTGGGTGAGAACGGTGTCT
mcr-1	CS1-mcr1721-68-for	ACACTGACGACATGGTTCTACAAAAGCTGTTTGATGTCACCGC
