# Supplementary material for: Characterization of Colistin-Resistant Escherichia coli Isolated from Diseased Pigs in France
Source: Front Microbiol. 2017 Nov 21;8:2278. doi: 10.3389/fmicb.2017.02278 (PMC5702452; doi:10.3389/fmicb.2017.02278)
Supplement: Table S2 — Disk diffusion, colistin MICs and presence of the mcr-1 gene in the isolates. [file Table2.pdf]

Table S2: Disk diffusion, colistin MICs and presence of the *mcr-1* gene in the isolates

Disk diffusion (NFU-47-107 disk diffusion method (AFNOR, 2012))	MIC (Sensititre)	Detection of *mcr-1*
81 isolates from the RESAPATH collection with colistin inhibition zone diameter **less than 17 mm**	79 isolates with colistin MIC > 2 mg/L: **CSTR** isolates	70 isolates with *mcr-1*<br>9 isolates without *mcr-1*
	2 isolates with colistin MIC = 2 mg/L: **CSTS** isolates	2 isolates with *mcr-1*
9 isolates from the RESAPATH collection with colistin inhibition zone diameter **higher than 17 mm**	9 isolates with colistin MIC ≤2 mg/L: **CSTS** isolates	9 isolates without *mcr-1*

CSTR: colistin-resistant

CSTS: colistin-susceptible
